# A Deep Learning Framework for Design and Analysis of Surgical Bioprosthetic Heart Valves

**DOI:** 10.1038/s41598-019-54707-9

**Published:** 2019-12-06

**Authors:** Aditya Balu, Sahiti Nallagonda, Fei Xu, Adarsh Krishnamurthy, Ming-Chen Hsu, Soumik Sarkar

**Affiliations:** Iowa State University, Department of Mechanical Engineering, Ames, Iowa 50011 USA

**Keywords:** Computational models, Machine learning, Valvular disease, Biomedical engineering

## Abstract

Bioprosthetic heart valves (BHVs) are commonly used as heart valve replacements but they are prone to fatigue failure; estimating their remaining life directly from medical images is difficult. Analyzing the valve performance can provide better guidance for personalized valve design. However, such analyses are often computationally intensive. In this work, we introduce the concept of deep learning (DL) based finite element analysis (DLFEA) to learn the deformation biomechanics of bioprosthetic aortic valves directly from simulations. The proposed DL framework can eliminate the time-consuming biomechanics simulations, while predicting valve deformations with the same fidelity. We present statistical results that demonstrate the high performance of the DLFEA framework and the applicability of the framework to predict bioprosthetic aortic valve deformations. With further development, such a tool can provide fast decision support for designing surgical bioprosthetic aortic valves. Ultimately, this framework could be extended to other BHVs and improve patient care.

## Introduction

Semilunar valves (i.e. the aortic and pulmonary valves) are structures that permit blood to be pumped into the aorta and pulmonary artery from the ventricles during systole, and prevent backflow into the ventricles during diastole^1^. Valvular heart disease is clinically characterized either by gradual narrowing of the valve due to calcification of the leaflets or regurgitation through the valve due to insufficient valve closure^2^. Valve repair and replacement are two possible interventions to address diseased valves and prevent congestive heart failure or death. Based on the estimates from the American Heart Association, more than 2.5% of the United States population is affected by valvular heart diseases^3^. Heart valve replacement is common for patients suffering from valvular heart valve disease; over 90,000 prosthetic heart valves are implanted in the United States every year^4^. One of the most popular classes of replacement valves are surgical bioprosthetic heart valves (BHVs), fabricated from chemically-treated biological tissues^2^. They provide better hemodynamic characteristics than mechanical prostheses (the other most popular class), but are prone to fatigue failure, limiting their durability to 10–15 years. However, estimating the remaining life of a BHV directly from medical images is difficult. On the other hand, valve performance measures can be used by physicians to make better valve replacement decisions, preventing premature replacements or surgeries^5,6^.

Computational analysis of the heart valve can be an important tool in understanding the etiology of valvular diseases and can help clinicians in obtaining additional information that aid in therapeutic or valve replacement decisions. For example, the failure of aortic valves can be related to stress concentration in the leaflets of the BHV^7^. Heart valve analysis using computational models has been extensively studied in recent years^2,7–11^. A review of computational modeling methods that have been developed to provide diagnosis from medical images for aortic valves is presented by Zakerzadeh *et al*.^9^. Several quantities of interest can be obtained and studied from these computational models. Two key quantities of interest are the coaptation area, which reflects of the degree of valve closure, and the effective orifice area (or open area), which reflects the degree of valve opening. The closure of the heart valve can be assessed by performing structural analysis of the valve geometry with appropriate boundary conditions to simulate the valve closure. Similarly, the effective orifice area can be computed by performing valve opening simulation or dynamic simulation of the valve for one complete cycle of the heart beat^9,10^. In this paper, we restrict our focus to valve closure simulations; however, our framework can be extended to dynamic simulations for obtaining other quantities of interest such as effective orifice area.

An accurate representation of the heart valve geometry is essential to assess its performance. Non-uniform rational B-splines (NURBS)^12^ have been the de facto standard for geometry representation in mechanical computer-aided design (CAD). NURBS surfaces can be used to parametrically design complex geometric objects, while allowing easy modifications. Current state-of-the-art valve analysis approaches reconstruct the aortic heart valve geometry from computed tomography (CT) images using NURBS^5,10^. In addition, current BHVs are designed only for certain discrete population-averaged sizes. These geometries are then analyzed using shell formulations of finite element analysis after meshing to assess the valve performance^5^. One of the most promising new analysis technologies that can be also used for valve simulations is isogeometric analysis (IGA)^13^. IGA unites engineering analysis and design by eliminating the tedious process of finite element mesh generation from the design geometry. IGA uses the B-spline basis functions for both representing the geometry and for the analysis. Hence, the NURBS valve geometry can be directly used for both valve design and analysis using IGA^10,14–17^.

**Figure 1:**
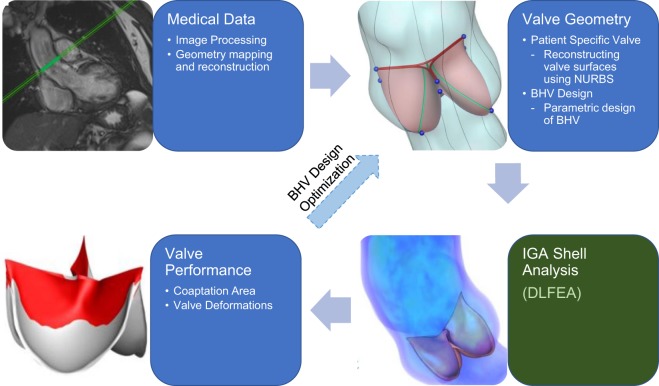
A framework for predictive biomechanics-based approach for design of BHVs. Evaluating the valve performance using finite element analysis is a critical time-consuming step in the process. DLFEA can replace compute intensive biomechanics simulations with fast valve performance evaluations.

The complete pipeline for the design and analysis of heart valves is illustrated in Fig. 1. While these analysis frameworks have proven to be useful, they often involve large computational overhead. Deep learning can provide a viable fast alternative to computational analysis, specifically IGA, to accelerate the design and analysis process for bioprosthetic valves. Deep learning has emerged as a major machine learning paradigm that has demonstrated transformative potential in many areas of science and engineering^18^. In sciences, the applications range from astronomy^19^, high-energy physics^20^ and material science^21–23^ to medical diagnostics^24^ and plant sciences^25^. In the domain of engineering, deep learning is the key enabler of the recent autonomous driving revolution^26^ along with other significant progresses in robotics^27^, design and manufacturing^28,29^ and prognostics and health management of engineered systems^30^. Along with recent algorithmic advances, the success of deep learning as a powerful function approximator could be attributed to the availability of large volume of data and advances in high-performance computing such as the Graphics Processing Units (GPUs).

In this work, we leverage the advances in deep learning to overcome the computational overhead in analysis of heart valves and to make valve design decisions. Note that in this work, we only specifically refer to surgical aortic valves with a stent that is sutured to the aortic root. Specifically, we propose a deep-learning-based convolutional autoencoder architecture (referred to as DLFEA) for predicting the analysis output directly from the input heart valve geometry. This approach accelerates the analysis by acting as a surrogate model that can be trained using previous analyses of multiple simulations. In particular, as a proof of concept, we predict the final deformed closed shape of the heart valve and the coaptation area, a key quantity of interest for surgeons. Coaptation area has been widely used as a key valve performance metric in the design and diagnosis of heart valves and predicting it has been the focus of several previous studies^5^. The stresses and strains during the heart valve closure can be directly computed from the heart valve deformations. However, the proposed methodology is general; it can be extended to analyze other key performance characteristics of heart valves and more complex valve simulations that include fluid structure interaction^10,15–17,31^.

We have the following specific contributions: (i) a deep learning framework, DLFEA, to predict the deformation biomechanics of aortic valves trained using isogeometric analysis simulation data; (ii) a novel geometric analysis tool called *NURBS-aware convolution* to directly input the valve geometry information to the deep-learning model; and (iii) statistical and anecdotal results that establish the accuracy and robustness of the proposed method. Please refer to the *Related Works* section in the Methods for a detailed discussion of our contributions in the context of recent advances in machine-learning based surrogate modeling for simulations. The results suggest that the DLFEA framework can be directly used in the design and optimization of patient-specific BHVs.

## Results

The DLFEA framework predicts the final deformations of the aortic valve and the valve coaptation area using the original undeformed geometry of the valve, the aortic pressure, and the material properties of the valve as input. We performed 90,941 valve closure simulations by varying the undeformed geometry, pressure, and material properties. Of these simulations, 72,753 simulations were used for training the DLFEA; 9,094 simulations for validating the training and hyperparameter tuning; and the rest for testing. We compare the performance of DLFEA framework on a test dataset (containing results from 9,094 simulations), which were not used for training.

**Figure 2:**
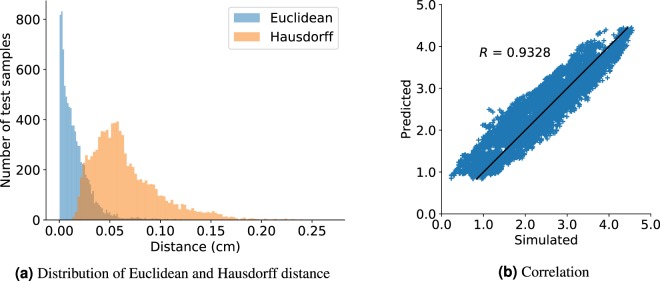
The histograms (**a**) show the Euclidean distance and Hausdorff distance between predicted deformations from DLFEA and the simulated deformations for the test data. (**b**) Shows the DLFEA-predicted coaptation area compared with the coaptation area obtained from simulations. The predicted coaptation area is highly correlated (*R* = 0.9328) with the simulated values.

The DLFEA framework can accurately predict the valve deformations. The valve deformations were compared using three metrics: Euclidean distance, Hausdorff distance, and Procrustes matching (see Table 1). The average Euclidean distance between the predicted and simulated valve deformations is 0.0649 cm (note the average diameter of a heart valve is 2.3 cm^32^). An histogram of the Euclidean and Hausdorff distances between the predicted and simulated deformed valve geometry is shown in Fig. 2(a). In addition, the DLFEA can also accurately predict the valve performance quantities of interest; in this case the coaptation area. The root mean squared error and the correlation between the predicted and the simulated coaptation area is 0.1167 cm^2^ and 0.9328, respectively (see Fig. 2b).

**Table 1:** Statistics on the metrics of deformations predicted by the DLFEA.

Stats	Euclidean			Hausdorff			Procrustes		
	Max.	Mean	Median	Max.	Mean	Median	Max.	Mean	Median
Training	0.1546	0.0173	0.0122	0.2908	0.0830	0.0779	0.0206	0.0021	0.0015
Validation	0.1337	0.0173	0.0120	0.2444	0.0827	0.0778	0.0173	0.0021	0.0014
Test	0.1502	0.0173	0.0119	0.2897	0.0821	0.0770	0.0204	0.0021	0.0014

All metrics are in cm.

**Figure 3:**
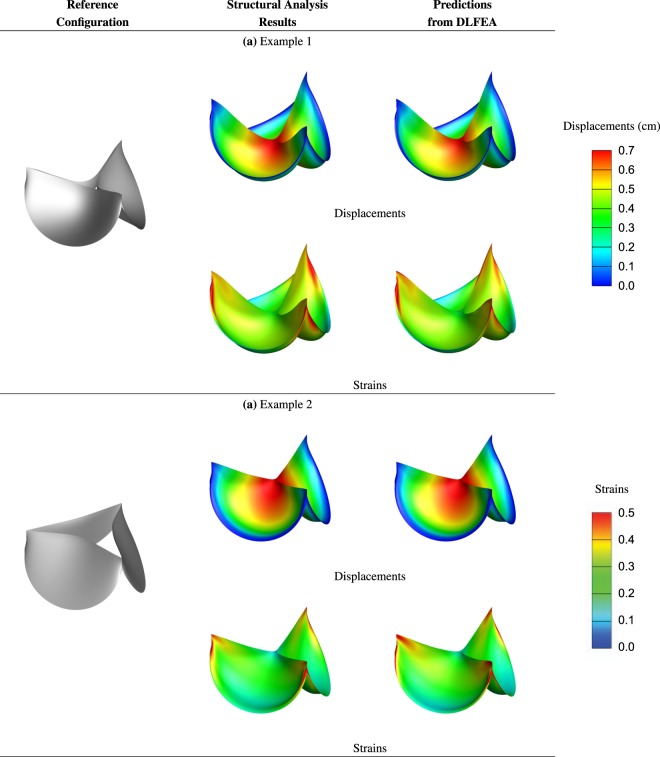
Illustrative examples of valve deformations and their corresponding maximum in-plane principal Green-Lagrange strains computed from isogeometric simulations and the predicted deformations using the DLFEA framework.

We present some anecdotal BHVs to understand the generalization capability of the trained model. We visualize the predicted and simulated deformed geometry of the leaflets and the strains computed using the simulated and prediced deformations in Fig. 3.

**Figure 4:**
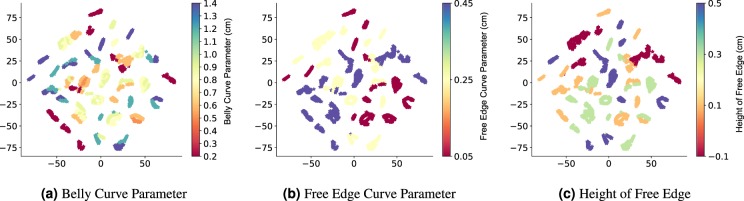
t-distributed stochastic neighborhood embedding (t-SNE) of the higher dimensional manifold learnt by DLFEA. t-SNE generates a lower dimensional embedding of the data using the learnt model, which can provide insights into the distribution of the data. This particular t-SNE shows that the different geometries are well clustered, showing that the model has reasonably learnt the effect of geometric parameters used for generating the reference configurations, although this information is not available to the model.

Finally, we also visualize the high-dimensional data manifold using t-distributed stochastic neighbor embedding (t-SNE)^33^. We perform this by analyzing the correlation between the learned information vector from DLFEA (the output of fusion layer in DLFEA, also called the code layer of an autoencoder) and the features of the input. In Fig. 4, we show the 2D embedding colored based on different geometric parameters (belly curve parameter, free edge curve parameter, and height of free edge, which were used to obtain different reference configurations) to understand the correlation between the data manifold and the valve geometry.

## Discussion

The results presented in the above section show that the DLFEA framework is able to accurately capture the deformation biomechanics to within reasonable error bounds. The maximum value of the displacement among all the valve leaflets from the simulations is 0.7642 cm. The maximum Hausdorff distance (which is the maximum among all measures) between the predicted and simulated valve displacements is within 15% of this maximum displacement. In addition, the median and mean value is less than 5% of the maximum displacement.

The predicted and simulated deformations are visually quite similar with some minor regional differences that are within ≈10% error, which is usually within the measurement accuracy of non-invasive imaging modalities such as Echocardiography^34^. In addition, the deformations predicted by DLFEA are accurate and smooth enough that the strains calculated from them are also smooth without any oscillations. More interestingly, we find that the DLFEA is able to learn certain complex interaction characteristics of the leaflets and their deformations. The predictions by DLFEA capture the symmetry of the three leaflets without any explicit training constraints. The DLFEA also captures the contact characteristics between the three leaflets accurately without any explicit information about contact mechanics (for example, penalty for interpenetrating leaflets).

The t-SNE embedding is the lower dimensional representation of the data manifold and if the network is properly trained, this embedding is the representation of the original data-manifold in a compact lower dimensional space that nearly preserves the distance metric^35^ (that is, similar input data are close in the t-SNE embedding). In the t-SNE embedding of our DLFEA network (Fig. 4), we see that this compact lower-dimensional space represents the complete variation in the data (design) space (i.e. the behavior of the valve deformation due to all the input parameters is well learnt). The local clustering of the training data in the t-SNE visualizations based on the geometric parameters demonstrate that the machine learning network has learnt the underlying geometric parameters of the valve. For example, in Fig. 4(a), similar belly curve parameter values are clustered together. Note that the geometric parameters are not provided as input to the t-SNE algorithm; it is only used for labeling the visualization of the points. In addition, none of the geometric parameters are directly given as input to DLFEA. The network is able to learn this relationship from the training data alone. Please refer to the Supplement for a more detailed study of the t-SNE based on the domain knowledge.

**Figure 5:**
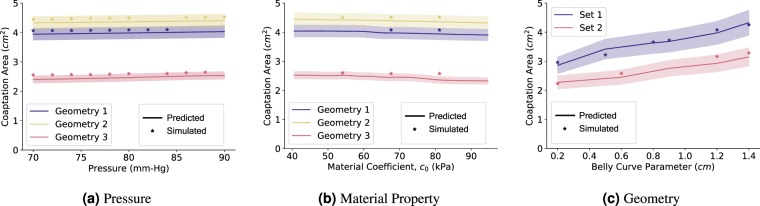
The DLFEA-predicted coaptation area variation with pressure is shown in (**a**) for three specific sets of reference configuration geometries. (**b**) Shows a similar plot with variation in material coefficient 1 (see Supplement for more details) for three specific reference configurations, pressure, and other material properties. (**c**) is a similar plot with variation in geometry parameter (belly curve parameter, see Supplement) for two specific material properties, and pressure. These plots are generated with 1000 intermediate values in the parameter of interest. The region estimating 10% variation of the predicted coaptation area value is highlighted.

The predicted coaptation area correlates highly with the simulated value (Fig. 2(b), *R* = 0.9328). In order to further understand the efficacy of DLFEA in interpolating and extrapolating the coaptation area predictions for different parameter values, we performed an ablation study (Fig. 5). We first fixed the reference geometry and material properties to vary the pressure (Fig. 5(a)) and calculated the predicted and simulated coaptation areas. Similarly, we fixed all the parameters including geometry and varied only one of the material properties (Fig. 5(b)). Finally, we performed a similar study for one of the geometric parameters (Fig. 5(c)). The predicted values are generated by densely varying the corresponding parameter in the physiological range (with 1000 intermediate values, for example, pressure is varied uniformly from 70 mmHg to 90 mmHg with an increment of 0.2 mmHg to generate 1000 data points). We also chose a few random parameter values and performed the valve closure simulations to compute the simulated coaptation area. This experiment was repeated for several parameter sets. In Fig. 5, we also highlight the region estimating 10% variation of the coaptation area value from the predicted values. The difference between the simulated and predicted values are within this 10% error margin. Moreover, the predicted values smoothly capture the overall trend of the change in the coaptation area with respect to the different parameters, without any artifacts. This demonstrates the generalization capability of the network for any parameter value in the physiological range. Such a generalization capability over parameters such as belly curve parameter is an interesting outcome from the hierarchical learning of deep learning from the raw geometric representation. Finally, this shows that such a system can be used as a fast function evaluator for an optimization system, which can be used to design optimal prosthetic valve geometries.

There are some limitations to the current implementation of the study. We do not account for anisotropy of the valve tissue material, which needs to be considered for more accurate modeling of the valve deformations. In order to make the study patient-specific, a large amount of patient data covering the geometry variation of aortic valve among a diverse set of population is needed. In addition, the BHV design parameter space needs to be validated to make sure that the resulting valve designs cover the complete geometry variation in the valves of these patients. Finally, new methodology that is accurate in handling cases with out-of-distribution samples while performing inference needs to be developed. These improvements would make the system practical for patient-specific valve design.

**In conclusion**, we have presented a deep-learning-based framework to predict the deformation biomechanics of heart valve that are not directly captured using medical imaging and often require elaborate computational expertise and cost to determine. We have demonstrated the capability of this methodology to learn complex deformation biomechanics of the heart valves with different geometry, material properties, and boundary conditions. This makes the framework directly useful for parametric design of BHVs. Such a fast decision support system can enable development of personalized heart valve designs with better fit and performance, ultimately improving patient care.

## Methods

Deep Learning, a subset of machine learning approaches, has emerged as a versatile function approximator that can establish a reliable map between (possibly heterogeneous) inputs and outputs of complex phenomena. A deep neural network is made up of several layers *l*_*i*_, which takes as input ${x}_{{l}_{i}}$ and produces an output ${y}_{{l}_{i}}=\sigma ({W}_{{l}_{i}}.{x}_{{l}_{i}}+{b}_{{l}_{i}})$, where *σ*(.) represents a non-linear activation function, ${W}_{{l}_{i}}$ and ${b}_{{l}_{i}}$ are the weights and biases, respectively, for connecting the input neurons to the output neurons. The connections could be as simple as a dense connection between every input neuron and output neuron. However, dense connections may fail to preserve local correlations in input that may encode useful information, for example, in the case of image classification. Furthermore, learning the dense connectivity between the neurons increases the sample complexity and all connections may not be meaningful. A convolutional connection instead of a dense connection helps alleviate these issues. The convolution operation ($\otimes$) is given by

1 $$W[m,n]\otimes x[m,n]=\mathop{\sum }\limits_{i=-h}^{i=h}\,\mathop{\sum }\limits_{j=-l}^{j=l}\,W[i,j]\,x[m-i,n-j].$$

Recently, deep learning has been successfully deployed in several areas with newer and more sophisticated architectures such as variational autoencoders^36,37^, generative adversarial networks^38^, graph convolutions^39^, etc. Once the network architecture is defined, the network weights (that are initialized randomly) are updated using the back-propagation algorithm^40^ based on minimizing a loss metric. At the end of training, the best weights and biases that generate the minimal prediction loss are chosen for the network. This network can then be used to predict the outputs for the test inputs.

### Related Works

Deep learning applications to create surrogate models for finite element analysis is a very recent area of research that specifically focuses on bridging physics based models and data-driven models. Some of the recent advances in this research area are summarized below:

1. **Physics-consistency in deep learning:** The overall idea is to merge the ideas of deep learning and physics by using physics-based features while training the deep learning models. For example, researchers have modified the loss functions to ensure some physical constraints are satisfied^41–45^. There has also been work on interpreting the predictions of the deep learning model based on physical conditions^22,23^.
2. **Incorporating partial differential equations** (**PDEs**) **in deep learning models**: The key idea is to use the underlying governing equations such as Berger’s equation, Navier-Stokes equation, Cahn-Hillard’s equation, etc. to compute the residual for the sample. Since modern software systems can define these partial differential equations numerically in terms of automatic differentiable functions, it is easy to minimize these residuals. There are several recent works on learning from partial differential equations^22,46–51^.
3. **Generative vs. distinctive predictions**: While there are methods in Deep Learning for generating and even predicting desired outputs, the underlying physics is often more strict. For example, given a set of physical conditions (such as loads on a well-defined geometry) will result in a deterministic desired output (such as displacements). Modeling them as a generative model is not consistent with the physics. On the contrary, the inverse problem, of defining the displacement of a given geometry and predicting the set of physics conditions is often ill-posed and could be consistently modeled as a generative model. There are several works showing the capability of Deep Learning methods to act as a surrogate^23,41,49,51^. These surrogates are modeled as distinctive (non-generative) networks, since the physics is deterministic and the problem is well-posed. On the contrary, there are some recent works^22,42,46^, which deal with stochastic PDEs or with ill-posed problems such as inverse design which demand the use of a generative model.

In the application area of biomechanics, most of the existing works use simple deep learning or physics-consistent deep learning methods. Specifically, these methods have been applied for modeling the aorta and estimating the stress fields^52–54^ or estimating the constitutive model parameters for aortic wall^55,56^. For BHVs, while there are optimization based methods for design of transcatheter aortic valves^57,58^, machine learning methods have mainly been used for 3D reconstruction of the valve geometry^59^. Further, there are physics-informed deep learning approaches for modeling cardiovascular flows, which incorporate residual minimization of the PDEs using deep learning^60,61^. However, to the best knowledge of the authors, deep learning has not been used for analyzing the deformation behavior of bioprosthetic valves.

In this work, we leverage the advances in deep learning to model the analysis of BHVs and to accelerate their design. The efforts made in physics-consistent deep learning and deep learning applications to biomechanics motivates this work. While there are several works in this fast growing area, there are still some gaps which are yet to be filled. This paper addresses some of those gaps:

1. **Predicting raw values vs. descriptors for biomechanics applications**: The current state-of-the-art machine learning works in biomechanics directly predict the stress field. However, during the physics solve, the stresses are not directly obtained. While it is challenging to obtain displacements from stresses, it is straight-forward to obtain the stresses and strains from displacements. Even though, small variations in the displacements can lead to large oscillations in the strain computations, in our case (see Fig. 3), the maximum principal strain obtained from predicted deformations is accurate and without any numerical oscillations. Therefore, we attempt to be consistent with the way physics is modeled to enable future work in using PDEs for computing residuals. Incorporating PDEs for modeling the complex dynamics involved in the bioprosthetic aortic valves is not a trivial extension of the present work, but, this contribution is a step forward towards that end.
2. **Contact prediction in deep learning**: Current physics consistent deep learning models and deep learning for biomechanics applications consider simple cases which doesnt involve interaction of multiple objects (or multiple features of the same object). This is important when we need to model contact physics among the objects. This is necessary in BHVs while predicting the contact between the leaflets. As seen in the Figures shown in Fig. 3, our method is able to learn the complex interaction of the three leaflets which is necessary while modeling the non-smooth behavior of the materials.
3. **Accurate representation of 3D geometries:** In general, there is a disconnect between the geometry, the physics domain mesh, and the data representation for training physics consistent deep learning model. Converting one form of data to another is computationally expensive and not accurate. To avoid this, often researchers use a structured mesh which could be expensive in case of representing geometries with complex geometric features such as the heart valve. On the contrary, we make use of a NURBS-aware convolution operation and isogeometric analysis to alleviate this issue.

### Deep-Learning for finite element analysis (DLFEA)

Learning the deformation biomechanics of heart valves involves learning multiple physical phenomena by the DLFEA framework. First, the DLFEA needs to learn from the input 3-dimensional Euclidean space geometry and predict the deformed shape also in 3-dimensional Euclidean space. Next, it needs to learn the effect of loads and boundary conditions on the deformation. It should learn about the interaction between the leaflets during closure (often dealt with by using a complex contact algorithm in traditional finite element analysis) to predict the coaptation area. Finally, it should learn the material behavior and the dependence of the deformation on the thickness of the leaflets used in the simulation.

Any machine learning framework requires the identification of three main components: (i) data representation, (ii) model architecture, and (iii) training algorithm. In the following subsections, we describe the different components of our DLFEA framework. Please refer to the supplement for details regarding parametric design of heart valves and simulation of valve closure using IGA. These methods were used for training data generation for the DLFEA framework.

#### Data representation

Learning directly from 3-dimensional Euclidean space is an interesting notion that has been explored extensively in machine learning literature. There are traditional approaches of object recognition using a 3D volume occupancy grid^62^ or its extensions such as Octrees^63^ and multi-resolution voxels^64^. Further, another class of algorithms have been developed to learn from point clouds^65^. There are other approaches where the topology of the data is modeled as a graph to perform a graph convolution operation^39^. However, the above mentioned methods have high sample complexity or time complexity for learning. Moreover, there is loss of information while transforming the CAD model to other representations. Hence, integrating the learning paradigm with the CAD representation is crucial. To this end, we propose a new NURBS-aware convolution operation.

**Figure 6:**
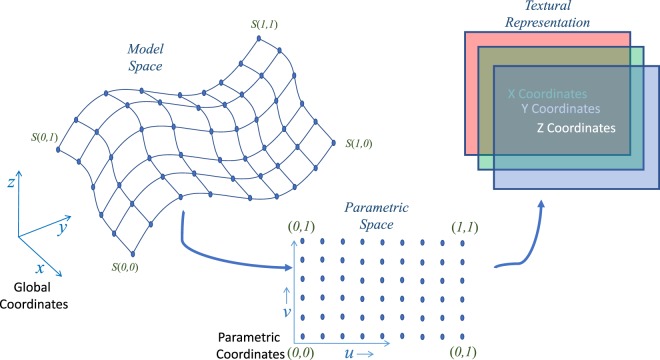
NURBS-aware convolution. In order to learn from a 3D surface, we extract the control points of the NURBS surface in the parametric space, which introduces spatial structure to the control points. We restructure the control points (from the parametric space) into three channels of an image (texture representation) to perform traditional convolution operations.

The crux of the NURBS-aware convolution operation is to perform the convolution operations on the NURBS control mesh of the input geometry to obtain the valve performance measures. The control points of NURBS surfaces provide the 3D surface representation of BHVs. Hence, for extracting the geometry information from different heart valves, it is sufficient to utilize the control points directly for the convolution operation. The control points can be represented as a rectangular matrix representing the tensor-product structure of these valve surfaces. Since, these control points are physically significant and are represented in the 3D Euclidean space, they can be represented as three different matrices with each of them containing the position with respect to each coordinate as shown in Fig. 6. This is equivalent to representing the control points as a RGB texture^66^. This method was used earlier to perform fast and parallel GPU evaluation of NURBS surfaces. Here, using this representation of the leaflet, we perform the convolution operation directly using the NURBS surface without any loss of information, and can learn using traditional convolutional neural networks. A similar mapping is required for deconvolving the final deformed geometry from the textural representation to the global coordinates.

#### Model architecture

**Figure 7:**
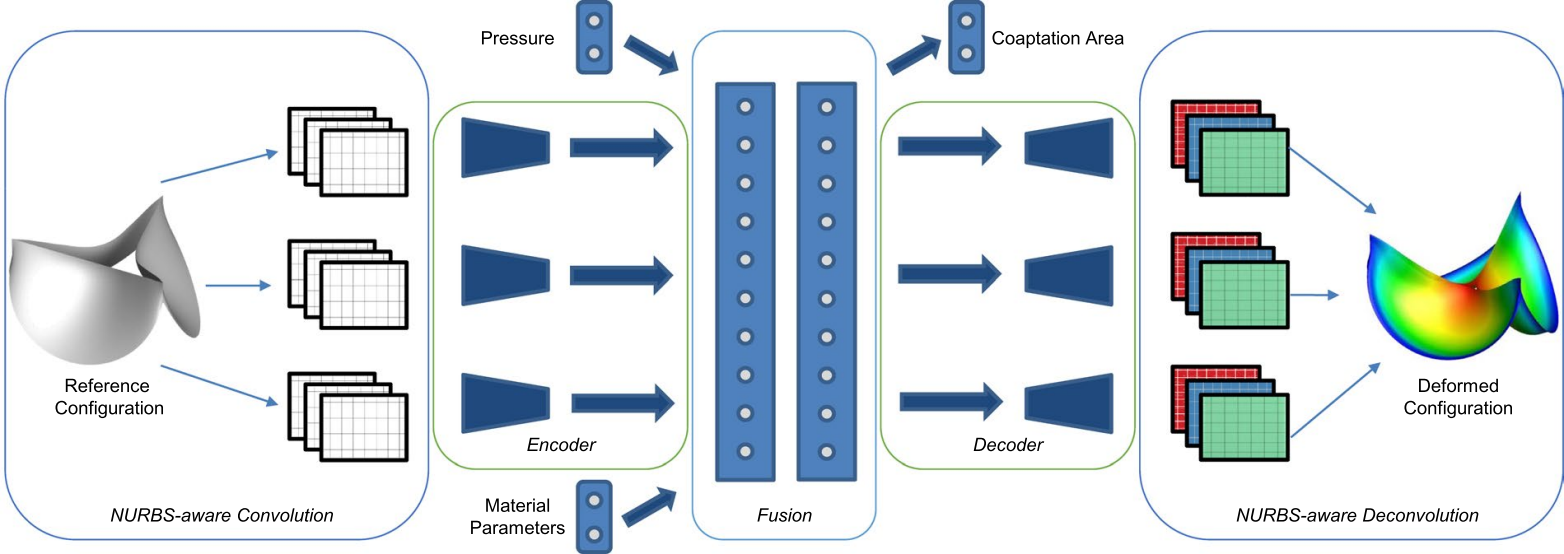
Deep-learning-based convolutional autoencoder for predicting the output deformations and the coaptation area of the heart valve in the closed state, with the BHV leaflet reference geometry, material properties, and the aortic pressure as input. The leaflet deformations are individually learnt using a NURBS-aware convolution followed by an encoder. All the inputs are fused using the intermediate fusion layers (also called as the coding layers).

We use convolutional autoencoder-type architecture to obtain the deformed geometry for a given heart valve. The autoencoder architecture is composed of three main components: (i) an encoder network, (ii) a code layer and (iii) a decoder network (see Fig. 7). An encoder network is designed to compress high dimensional input data into a lower dimensional embedding. The dimension of the embedding is very important since while a smaller dimensional embedding represents a succinct noise-less representation of the inputs, it also leads to a greater loss of information. This low dimensional embedding is represented in the code layer. Using this, one could generate/reconstruct the original high dimensional representation using a decoder network. An end-to-end training using the reconstruction loss is used to train the entire autoencoder. This method has been shown to quite effective for denoising and enhancing images and videos for computer vision^37,67^.

We split the heart valve into three leaflets and for each leaflet, we use the NURBS-aware convolution operation where the NURBS surface is represented using three rectangular matrices (of size *m* × *n* × 3, where *m* and *n* are the number of control points in both directions of the parametrization). Now, using this data representation, we convolve further using traditional convolutional layers to create the encoder block of the autoencoder^36,37^.

Thus, using an encoder, we represent the high dimensional surfaces to an informative lower dimensional representation. We then fuse the information of features obtained from the encoder for the three leaflets by flattening the low-dimensional output and connecting all the outputs using a fully connected layer. This is required to learn the interaction between the three leaflets, such as contact or closure of the leaflets. However, this increases the sample and time complexity of training due to the increase in the number of weight parameters. However, since we perform this operation in the low dimensional manifold, the increase in complexity can be accommodated efficiently. Ensuring the correct size of the code layer of the encoder is necessary, since there is a trade-off between complexity and performance. We vary the size of the code layer until we get the best performance for a set of samples.

Apart from the interaction, the deformation biomechanics depends on the material properties of the heart valve which are also used in the simulations. Uniform pressure is applied on the heart valve as a boundary condition. At the closed state, the valve is in a hydrostatic state of stress, where the loading is uniform in all directions. Hence, a scalar pressure value is sufficient to correctly define this boundary condition. However, in order to ensure that the scalar plays significant role in learning, we repeat the scalar pressure value multiple times to form a vector of size 10 (obtained after experimentation). We fuse the material properties vector and pressure boundary condition of the heart valve (pressure vector) with the fused embedding (fully connected layer obtained earlier) of the leaflet geometry.

Since the coaptation area is an important functional parameter used to determine the BHV health, we chose to predict this quantity of interest directly using the network. We also chose to predict the final deformed shape of the leaflet geometry, which can be used to obtain any other measures such as leaflet strains and also provide visual feedback of the deformed shape. The deformed geometry is predicted by using a decoder block which decodes the information from the fully connected layers used for fusing the information from thickness, pressure, and leaflet geometry. The decoder predicts the final deformed control points of the heart valve, which can be multiplied directly with the weights and knot vectors of the original geometry to get the deformed surfaces.

The final requirement for effective learning is to introduce a linear/non-linear activation for each output. In the case of the coaptation area, a rectified linear unit (ReLU) is the best fit, since the coaptation area is always non-negative. The ReLU function is represented as follows:

2 $${Re}LU(x)=\,{\max }(x,0).$$

On the contrary, deformations of the leaflets could be negative at some locations, which makes ReLU a bad fit to use as an activation function for this output. Hence, linear activation function is used for the deformations. In the next subsection we outline the details of training the proposed network architecture.

#### Training algorithm

In the previous two subsections, we explained the embedding of the different physical attributes in the data representation and the machine learning model to enable effective learning. This is required for effective learning since the sample complexity and time complexity are still a challenge. Although compute capability is abundant especially with the advent of GPUs, there are still limitations on the amount of data that can be generated in a viable time frame, specifically when the data is generated from computationally heavy simulations. Hence, embedding the physical attributes in different possible ways is required for effective machine learning methods. In addition, this helps us leverage some of our physical understanding of the process, thereby reducing the learning complexity for the machine learning network.

Another important physical characteristics that we need to embed are the essential boundary conditions imposed on the geometry for valve closure. Fixed boundary conditions affect the deformation of the BHV simulations. While the fixed node has zero deformation, achieving zero predicted deformation up to an arbitrary precision is numerically difficult. Further, if the back-propagation algorithm tries to achieve that, the nodes with non-zero deformation are affected, making the overall deformation difficult to learn. We deal with fixed boundary conditions by weighing the loss with the true deformations. If the original loss function used is *l*, the modified loss function is

3 $${l}_{bc}=\text{abs}(\frac{{u}_{true}}{\text{max}(\text{abs}({u}_{true}))})\,l,$$

where *l*_*bc*_ represents the boundary condition incorporated loss function and *u* represent the displacements. In practice, we use *l* to be mean squared error:

4 $$l=\frac{1}{|{\mathscr{D}}|}\,\sum _{k\ast {\mathscr{D}}}\,{({u}_{pre{d}_{k}}-{u}_{tru{e}_{k}})}^{2},$$

where ${\mathscr{D}}$ is the dataset to learn from, $|{\mathscr{D}}|$ is the number of data points used for training and *u*_*pred*_ and *u*_*true*_ represent the predicted and true displacements respectively.

In order to improve the sample complexity, we also perform data augmentation^68^. Using arbitrary scalar values, we shift the BHV models to generate modified control points of a new BHV, which augment the original training data. We make use of a parametric BHV design algorithm (see Supplement) to generate different valve geometries. These valve geometries, along with different valve material properties and aortic pressures, are used to simulate valve closure using isogeometric analysis. Part (60%) of the simulated deformed geometries along with their calculated coaptation area are used as data for training the DLFEA framework. Of the remaining data, 20% was used for validation and 20% for testing.

## Supplementary information

Supplementary information

Video demonstrating our results

## Data Availability

The datasets generated and/or analysed during the current study are available at http://web.me.iastate.edu/idealab/c-dlfea.html.
