# Supplementary material for: A Deep Learning Framework for Design and Analysis of Surgical Bioprosthetic Heart Valves
Source: Sci Rep. 2019 Dec 6;9:18560. doi: 10.1038/s41598-019-54707-9 (PMC6898064; doi:10.1038/s41598-019-54707-9)
Supplement: Supplementary file 1 — Supplementary information [file 41598_2019_54707_MOESM1_ESM.pdf]

A Deep Learning Framework for Design and Analysis of Surgical Bioprosthetic Heart Valves

Supplementary Material

Aditya Balu, Sahiti Nallagonda, Fei Xu, Adarsh Krishnamurthy*, Ming-Chen Hsu, Soumik Sarkar
Iowa State University, Department of Mechanical Engineering , Ames, 50011, US

NURBS-based modeling of surfaces

Non-Uniform Rational B-spline (NURBS) surfaces are the most general parametric representation of smooth surfaces. NURBS surfaces are represented using a set of weighted control points and two knot vectors, one for each parametric direction, u and v. The knot vectors control the parametric spacing between the control points. Intuitively, the NURBS surfaces can be thought as a smooth surface approximation guided by the control points, whose continuity is controlled by the knot vectors, and the relative importance of the control points by the weights.

Mathematically, NURBS are a generalization of B-splines. The NURBS surface is defined by a $m \times n$ control points mesh, $P_{i,j}$ with corresponding weights $w_{i.j}$; two parametric directions, u and v with their associated knot vectors, degrees (p and q), and basis functions, $N_{i,q}(u)$ and $N_{j,q}(v)$. The knot vector is a set of parametric coordinates which divide the B-Spline into piecewise sections. If the knot intervals are equal, the spline is considered uniform. The basis functions are defined using the Cox-de Boor recursion formula[9]:

$$N_{i,p}(u) = \frac{u - u_i}{u_{i+p} - u_i} N_{i,p-1}(u) + \frac{u_{i+p+1} - u}{u_{i+p+1} - u_{i+1}} N_{i+1,p-1}(u) \tag{1}$$

with

$$N_{1,p}(u) = \begin{cases} 0, & \text{if } u_i \leq u \leq u_i + 1 \\ 1, & \text{otherwise} \end{cases} \tag{2}$$

NURBS surfaces are a generalization of B-spline surfaces with the addition of weights, $w_{i,j}$, assigned to each control point, $P_{i,j}$. The basis functions are modified to be

$$R_{i,j}^{p,q}(u,v) = \frac{N_{i,p}(u) N_{j,q}(v) w_{i,j}}{\sum_{k=0}^{n} \sum_{l=0}^{m} N_{k,p}(u) N_{l,q}(v) w_{k,l}}. \tag{3}$$

The NURBS surface is defined as:

$$S(u,v) = \sum_{i=0}^{n} \sum_{j=0}^{m} R_{i,j}^{p,q}(u,v) P_{i,j}. \tag{4}$$

Learning from NURBS representation

Any machine learning framework requires careful selection of three main components: (i) data representation, (ii) model architecture, and (iii) training algorithm. For effective learning, the domain knowledge needs to be embedded in each component. In convolutional neural networks for image recognition, one embeds the spatial localization using the convolution filters (model architecture) and the pixels of the image are represented using multiple red-green-blue-alpha channels (data representation)[6,7]. For design for manufacturing application, embedding the CAD models in a voxel-based representation was required to learn the volumetric features using 3D convolution filters (model architecture)[3]. Note that sophisticated model architectures are not essential to train the machine learning network in these problems; these could be trained by using basic dense neural networks. However, the data and time requirements for learning such dense network increases combinatorially. Therefore, the chosen approach of NURBS-aware Convolution is necessary for effectively learning from the NURBS CAD representation. A simple demonstration of

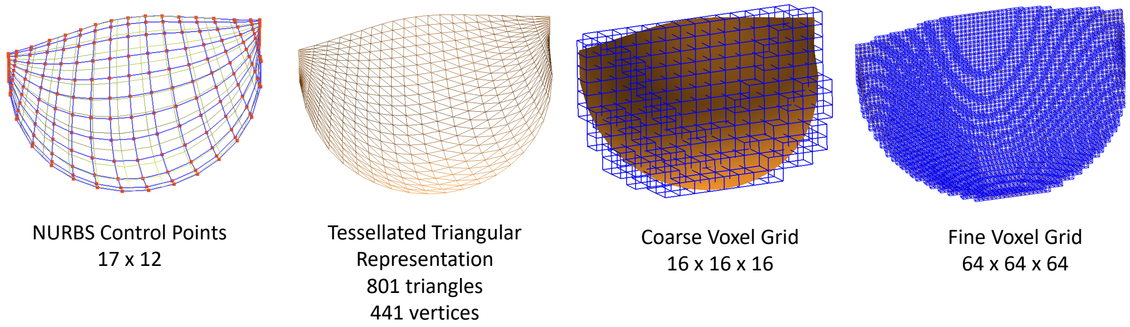

Figure A1: *The different representations of a leaflet of the heart valve and their dimensionality. The representation using the control points is the most succinct and sound representation among all.*

the effectiveness is shown in Figure A1. It can be easily understood that the dimensionality of the NURBS-aware convolution operation is lower than other representations and hence the data requirements are also lower. At the same time, any representation other than the direct usage of control points also involves some loss of information. For example, a coarse level voxel grid may not be able to capture all the features of the leaflet. However, a lossless representation of the geometry is necessary for understanding several key notions of biomechanics, such as contact. A fine level voxel grid might be a possible candidate; however, the dimensionality increases by a factor of ≈ 500.

In addition, data augmentation is necessary[10] for successfully learning the network weights. There are other approaches where the phenomena can be embedded in the algorithm to enable faster learning. In this study, we exploit these approaches of embedding the physical valve characteristics in the machine learning model to learn the deformation mechanics of heart valves.

Parametric design of heart valve

The aorta is the largest blood vessel that carries blood from the heart to the rest of the body. The aortic valve–one of the four heart valves–is located between the left ventricle and the left aorta. The aortic root represents the connection between the aorta and myocardium: it consists of the sinuses, the aortic valve leaflets, the commisures, and the interleaflet triangles. The three leaflets (left coronary leaflet, right coronary leaflet and non-coronary leaflet) form the aortic valve and provide its main sealing mechanism. The anatomy of the BHV leaflet can be divided into three parts, first, the free edge which provides sealing by forming contact with the neighboring leaflets to form a seal (the area of contact is called the coaptation area). Second, The "belly" of the leaflet and third, the bottom parts of leaflet or leaflet attachments. The aortic valve leaflets form the junction and physical boundary between the left ventricle and aorta.

Surgical BHVs considered in this work are fabricated from bovine and porcine pericardium sheets that are chemically fixed *after* being die-cut and mounted onto a metal frame to form the leaflets. As a result, the geometry shown in Figure A2 is without internal stresses and can be used directly as the stress-free configuration. The leaflet is initially flat in 2D before being mounted onto the metal frame. However, the non-flat leaflet in 3D represents the working condition of the BHVs and the analysis is performed using this configuration. In order to find the flat leaflet that can deform and then perfectly match the designed 3D shape, one can perform an inverse design simulation to reverse the process and iteratively find the 2D flat shape from the 3D shape.

The parametric design of a heart valve is divided into two steps; step (i) we define the size of the valve for a given aortic root, and step (ii) we define the shape parameters of the heart valve that define the geometry without any dependency on the size of the aortic root. The valve leaflets are parametrized using the aortic root as basis, First, 9 key registration points located on the ends of commisure lines at the bottom of the sinuses are identified. These define the attachment points of the leaflets to the sinuses, indicated by blue spheres in Figure A2. These key points are derived from the patient specific aortic root and will not change for different valve designs. The univariate B-splines are parametrized to define the free edges and belly

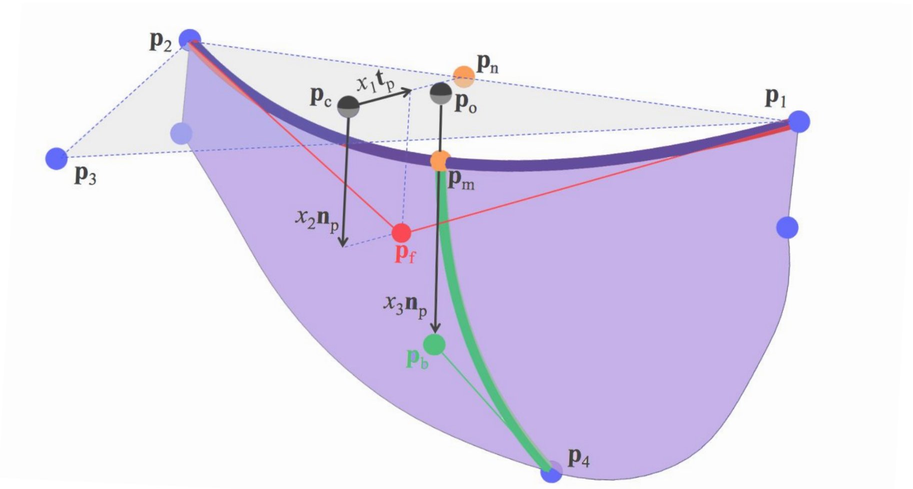

Figure A2: *Parametric design of bioprosthetic heart valves.*

curves of the leaflet, shown in red and green respectively in the figure. A smooth B-Spline representation of the leaflet is obtained by interpolation of the attachment edges, free edges, and belly curves. In Figure A2, p_1, p_2, p_3 are the key points on the top of commisure line and p_4 is the key point on the bottom of the sinus. P_1 to p_3 define a triangle with p_c being its geometric center. t_p is the unit vector pointing from p_c to p_n (geometric center of p_1 and p_2), and the unit normal vector of the triangle p_{1-3} pointing downwards in n_p. The free edge is constructed as a univariate quadratic B-spline curve determined by 3 control points, p_1,p_f and p_2. p_f is defined as $p_c + x_1 t_p + x_2 n_p$. By changing x_1 and x_2 to control the location of p_f, the curvature (length) and the height of the free edge can be parametrically modified. We then take p_m as the midpoint of the free edge, the point p_b, and the key point p_4 to construct a univariate quadratic B-spline curve (green). In this model, x_1, x_2, and x_3 can be chosen as design variables to parametrically change the free edge and belly curve and therefore, parametrically control the valve design. This procedure is implemented in Rhino/Grasshopper refer to Xu et. al.[12] for more details of parametric model.

Isogeometric analysis

The generation of data for training the DLFEA is performed using isogeometric shell analysis. Isogeometric analysis (IGA)[4] is an extremely useful tool in analyzing NURBS-based geometry by extending the finite element formulation to use the same NURBS basis functions for the analysis. The major advantage of IGA over traditional finite element method is that it does not require the geometry to be meshed, which is both tedious and causes loss of information due to mesh approximation. IGA has been proven to be useful for valvular analysis by several researchers[5,8].

Formulation

We perform dynamic simulations of pericardial BHV function at its closure with a prescribed transvalvular pressure load. The dynamic simulations are performed using Kirchoff-Love shell theory and Lee-Sacks material constitutive model (isotropic) (explained below). With the prescribed material properties and transvalvular pressure, we perform dynamic simulations while modeling the viscous and inertial resistance of the surrounding fluid using damping[5]. We perform the valve closure simulations by slowly ramping up the pressure to the prescribed transvalvular pressure in 100 time steps with a time step size of 0.0001 s. Then, with constant pressure, we run more steps till a steady-state valve closure is achieved. The damping coefficient is kept very high in order to quickly stabilize the ramp up of pressure. In the context of this paper, we perform valve closure simulations using structural simulations. Since the valve is closed, the structural analysis of heart valves will model the behavior very well (i.e. a hydrostatic state of stress exists and there is no flow). More advanced study of the behavior of heart valve may require fluid-structure interaction (FSI) simulations which involves temporal dynamics of blood flow for the complete cycle of the heart.

Material parameters

Kiendl et al.[5] built a generic Kirchoff-love shell theory for materials of this type and also provide a case study with an exponential-type isotropic model for strain energy function, ψ_{el}.

$$\psi_{el} = \frac{c_0}{2}(I_1 - 3) + \frac{c_1}{2}(e^{c_2(I_1-3)^2} - 1) \tag{5}$$

Here, I_1 is the first invariant of the right Cauchy–Green deformation tensor, $\mathbf{C}$. The magnitude of the parameters c_0, c_1, c_2 depends on the chemical treatment for the BHVs. The material is assumed to be incompressible, which is done by augmenting the elastic strain-energy function, ψ_{el} with a constraint term to enforce $J = \sqrt{det(\mathbf{C})} = 1$, via a Lagrange multiplier $p : \psi = \psi_{el} - p(J-1)$. In addition, the thickness of the leaflets is another key material property that changes the deformation behavior.

Dynamic simulations and convergence

While the default parameters for damping, time steps, and time step size, are as mentioned above, insufficient damping can lead to a large residual while ramping up to a particular prescribed material properties or transvalvular pressure from the reference configuration of the geometry. In addition, insufficient damping can also cause oscillations in the deformed geometry, leading to oscillations in the coaptation area and contact. Therefore, successful convergence of a particular simulation depends on carefully adjusting the parameters such as damping coefficient, time steps, time step size, etc. While generating the samples for training, since these parameters are dependent on each simulation, a generic set of parameters that would work for most of the simulations is used as default; specific cases of non-convergence are manually addressed by changing these parameters.

Mesh convergence

While the convergence of the individual simulations is important, the mesh resolution used for generating the simulation results is also important. In order to have an accurate solution for the valve deformation and to compute the coaptation area accurately, we performed a study on the convergence of the coaptation area with mesh resolution. we performed this study by choosing a representative bioprosthetic aortic valve with known geometry parameters and obtained different mesh representations of the NURBS surface to be used for isogeometric analysis. Figure A3 shows the computed coaptation area for different number of cubic elements corresponding to the mesh used for analysis. Based on this, we chose to create a representative NURBS patch using 17×12 control points for each leaflet. We observe that the coaptation area for this representative geometry in the chosen mesh resolution is $1.0116\,\text{cm}^2$, while the value for a finer resolution is $1.0541\,\text{cm}^2$. There is a 4% deviation in the coaptation area computed using our chosen resolution with respect to the finer resolution. This deviation in the coaptation area is acceptable, which balances the computational time with simulation accuracy. While increasing the resolution might improve the accuracy in the biomechanics simulations, it also increases the time taken for generating the data and simulations. Therefore, we use the chosen resolution for generating the data.

Data generation

The inputs to the DLFEA are (1) reference geometry, (2) the transvalvular aortic pressure and (3) material properties (c_0, c_1, c_2, thickness). Varying them to cover all kinds of material variations and all kinds of physiological conditions of different patients is necessary. Therefore, we use the list of thicknesses in the physiological region by choosing thicknesses provided in Section 3.1 of Caballero et al.[2]. Similarly, we vary other material properties by choosing the physiologically prescribed value given in Wu et. al.[11] and then varying them from 80% to 120% of that value. The geometries are obtained by changing the parameters provided in Table A1. Several simulations were run with different values for the valve thickness, material properties, aortic pressure, and geometric parameters such as belly curve parameter, height of the free edge, etc.

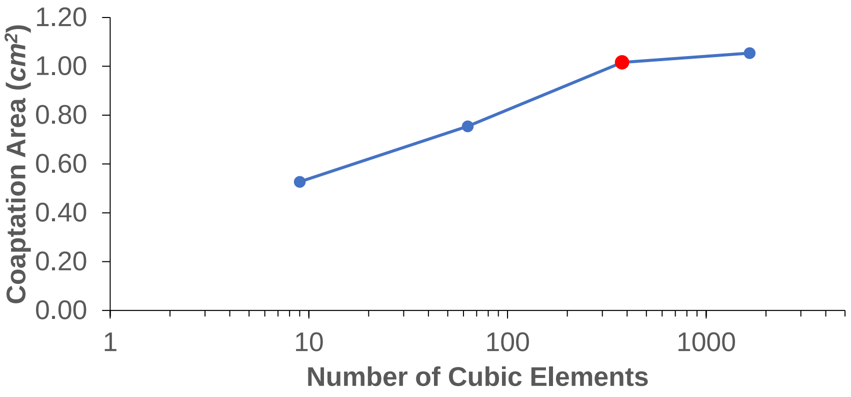

Figure A3: *Study of the convergence of the coaptation area with mesh resolution for a representative bioprosthetic aortic valve. The mesh resolution chosen for DLFEA data generation is marked in red.*

Parameter	Min. Value	Max. Value	Number of Values
Free Edge Curve Parameter (cm)	0.05	0.45	3
Belly Curve Parameter (cm)	0.2	1.4	7
Height of the Free Edge (cm)	-0.1	0.5	4
thickness (mm)	0.186	0.427	5
c_0(kPa)	54.084	81.130	3
c_1(kPa)	10.628	15.942	3
c_2	30.554	45.826	3

Table A1: *Parameters used for generating the reference geometries and material parameters required for training.*

Training

A total of 90,941 simulations converged with the default simulation parameters and were used for the training of the network. From the total data samples generated, a part of data is reserved for validation and for testing of the model for its generalization capability. The validation data is used to tune the hyperparameters of the network, where we optimize the number of convolution layers, number of fully-connected layers, number of channels in each of the convolution layers, number of neurons in each fully connected layer, etc. Using the validation data, we could ensure that we choose the best hyperparameters that produce the least

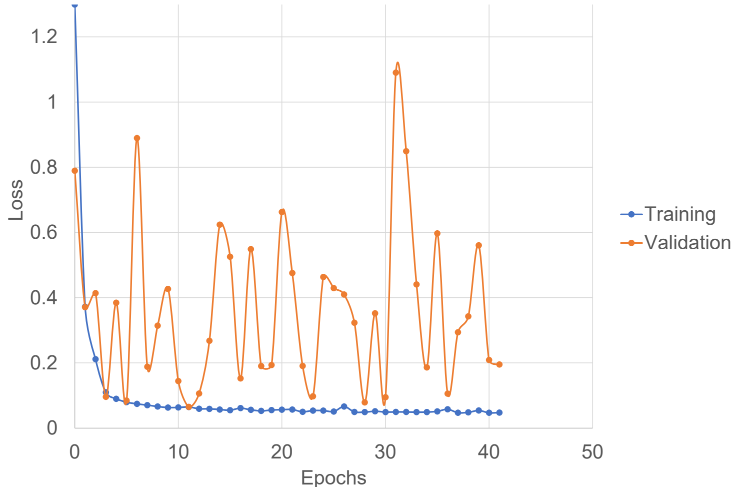

Figure A4: *Training and validation loss charecteristics with respect to epochs. The weights with minimum validation loss are stored for performing inference and other tests and visualizations.*

Common Training Parameters	DLFEA Architecture Block	Hyperparameters
Batch Size: 512<br>Optimizer: Adam	Encoder	Convolutional layer 1 (3 filters with size 5×5)<br>Convolutional layer 2 (8 filters with size 3×3)<br>Convolutional layer 3 (16 filters with size 3×3)<br>Convolutional layer 3 (32 filters with size 3×3)
	Repetitions of scalars	Pressure: 20<br>Thickness: 20
	Code layer	Fully connected layer 1(64 neurons)<br>Fully connected layer 2(48 neurons)
	Decoder	Deconvolution start size ($8 \times 9 \times 4$)<br>Deconvolution layer 1 (8 filters with size 5×5)<br>Deconvolution layer 2 (6 filters with size 3×3)<br>Deconvolution layer 3 (6 filters with size 3×3)<br>Deconvolution layer 4 (4 filters with size 3×3)<br>Deconvolution layer 5 (3 filters with size 1×1)

Table A2: *Optimized hyperparameters with the least validation loss.*

loss with not just the training data, but also with the validation data. However, to be fair in evaluating the performance of the model, it is a good practice to have a test of the performance on data that is not used for training and hyperparameter optimization. This proves the generalization of the data over the complete range of the input cases.

The training and validation loss variation with epochs for training is shown in Figure A4. In general, we save the weights of the model with least validation loss. We run for 30 additional epochs to check if the loss reduces further. In the Figure A4, we see that at the 11th epoch the training and validation loss are very close to each other and validation loss is minimum. Yet, we still run for 30 more epochs to ensure that the

Algorithm 1: Training Algorithm

Input : Network Architecture
Initialize: Weights for all layers, W_l, $(l = 1, 2, \ldots, m)$; patience = 0
Load Data: Load training data $\mathcal{D}$ and validation data $\mathcal{D}_V$
for $(i = 0; i \leq num\_epochs; i++)$ **do**
  Randomly shuffle the data
  Split $\mathcal{D}$ to $\mathcal{D}_j$, $(j = 1, 2, \ldots, n)$ mini-batches
  for $j = 1 : n$ **do**
    Predict outputs $\mathcal{O}_j$ for mini-batch $\mathcal{D}_j$
    Compute loss $\mathcal{L}(\mathcal{D}_j, \mathcal{O}_j, \{W\})$
    Update weights,$\{W\}$ using Adam optimizer
  end
  Predict validation outputs $\mathcal{O}_V$ for $\mathcal{D}_V$
  Compute Validation Loss$\mathcal{L}(\mathcal{D}_V, \mathcal{O}_V, \{W\})$
  if *Avg. Validation Loss not improving* **then**
    increment patience
  end
  else
    patience = 0
  end
  if *patience* $\geq$ *30* **then**
    Exit
  end
end

weights obtained are truly minimal and has good generalization capability. Finally, we stop at the end of 42 epoch because we do not find any better weights with a lower validation loss. The sum training loss for all the predicted outputs is 0.0594 and the validation loss is 0.0495 for the plot shown. The corresponding test loss is also of the same order due to the model having a good generalization capability. The final hyperparameters of the network (i.e. the number of convolution layers, number of convolution filters, the filter size, the code layer size, etc.) are shown in Table A2. The overall training procedure for a given set of hyperparameters is shown in Algorithm 1.

Statistical analysis

Error measurements

Two major metrics were used while comparing the results: (i) the root mean-squared error, and (ii) the correlation coefficient. The root mean-squared error is computed by:

$$RMSE = \sqrt{\frac{\sum_i^k (p_1 - p_2)^2}{N}}$$

The correlation coefficient (more popularly known as Pearson Correlation Coefficient, R), which was used for the comparison of the results is given by:

$$R = \frac{cov(x, y)}{\sigma_x \sigma_y},$$

where $cov(x, y)$ is the covariance between x and y and σ is the standard deviation.

A simpler formula used for computing is as follows:

$$R = \frac{\sum (x - m_x)(y - m_y)}{\sqrt{\sum (x - m_x)^2 \sum (y - m_y)^2}}.$$

Here m_x and m_y represent the mean of vectors x and y.

Distance measures between two 3D objects

Euclidean distance between two points p, q in N-Dimensional space is given by

$$d(\mathbf{p,q}) = \sqrt{(p_1 - q_1)^2 + (p_2 - q_2)^2 + \cdots + (p_N - q_N)^2}$$

By distance between two CAD representations, we refer to the mean of the euclidean distances of samples points in the surface of the representation. i.e. For a NURBS representation, we sample the points (say, M) for by varying the parameters u, v and evaluate the surface representation to obtain a set $\mathcal{P} = \{P^1, P^2, P^3, \ldots P^M\}$. Similarly, we could obtain another set $\mathcal{Q} = \{Q^1, Q^2, Q^3, \ldots Q^M\}$ for the other object. Distance Measures are made using the sets $\mathcal{P}$ and $\mathcal{Q}$. Note that, $|\mathcal{P}| = |\mathcal{Q}| = M$.

Euclidean distance

We define the Euclidean Distance to be the mean of the euclidean distances of each point in set $\mathcal{P}$ and set $\mathcal{Q}$. Mathematically, we can represent it as follows:

$$\mathcal{D}_{euclidean}(\mathcal{P}, \mathcal{Q}) = \frac{1}{M} \sum_{i=1}^{M} d(P^i, Q^i)$$

Hausdorff distance

Directed Hausdorff distance is the maximum of all the minimum distances from the set of points $\mathcal{P}$ to $\mathcal{Q}$. Symmetric Hausdorff distance is the maximum of the two directed Hausdorff distances. It represents the maximum possible deviation between two sets. Mathematically, symmetric Hausdorff distance is represented as follows:

$$\mathcal{D}_{hausdorff}(\mathcal{P},\mathcal{Q}) = max\{\sup_{P\in\mathcal{P}} \inf_{Q\in\mathcal{Q}} d(P,Q), \sup_{Q\in\mathcal{Q}} \inf_{P\in\mathcal{P}} d(P,Q)\}$$

Procrustes matching

Procrustes matching is a statistical tool particularly established to compare two geometric shapes while accounting for translation, rotation, and scale between them. Procrustes matching provides a metric of dissimilarity between two 3D geometries. Mathematically, the dissimilarity measure is:

$$\mathcal{D}_{procrustes}(\mathcal{P},\mathcal{Q}) = \frac{1}{M}\sum_{i=1}^{M}\sqrt{P^i - \beta Q^i \tau - \mathbf{1}\gamma},$$

where β, τ, γ are the similarity parameters representing the scale factor, rotation matrix and translation shift between the two shapes. These parameters are computed using the soft-assign Procrustes algorithm, which computes the rotation angle by taking the estimates of the first and second order moments of the data. $\mathcal{D}$ is known as the Procrustes dissimilarity measure[1] and it has the same units as the geometry. It is the residual obtained after modifying the data using the soft-assign Procrustes algorithm. The goodness of the fit is evaluated using the sum of squared errors criterion.

Additional results

The main paper shows the results for the variation of the coaptation area with material properties, pressure, and geometry. On careful examination of the variation of the coaptation area with respect to the material properties, we notice that there is not much variation of the coaptation Area with the material coefficients c_0, c_1, and c_2, in the physiological range of these parameter values (see Figure A5). Therefore, we come up with another refined network with just pressure, thickness, and reference configuration as the input to DLFEA as shown in Figure A6.

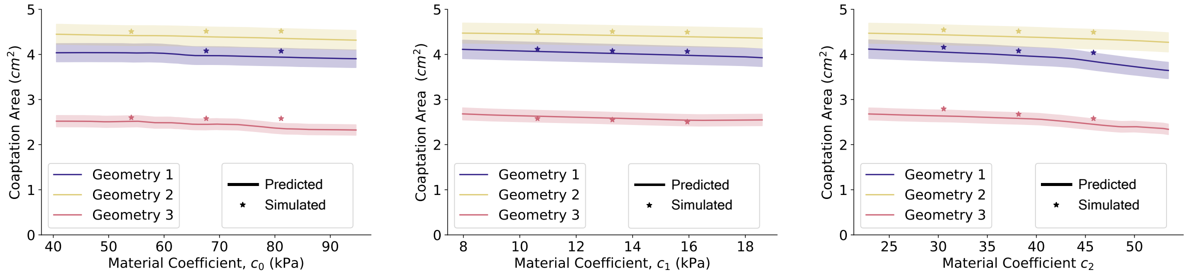

(a) c_0 **(b)** c_1 **(c)** c_2

Figure A5: *DLFEA-predicted coaptation area variation with the variation of different Material Coefficients. The highlighted region show 10% variation in the predicted coaptation area values.*

Alternate model with fixed material parameters

As explained in the previous section, there is not much variation in the coaptation area with the material properties. Due to this observation, we explored another model with fixed material properties. However, note that, material properties does play a crucial role in the several other quantities of interest, therefore, the original model proposed is more generic and can be used to generalize for other quantities of interest. The architecture of the modified network is shown in Figure A6. The training was performed with a subset

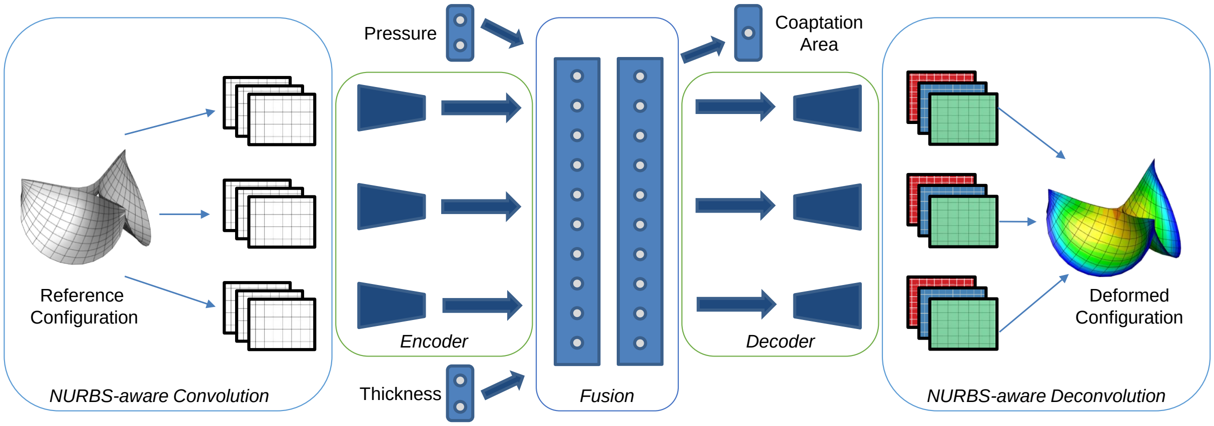

Figure A6: *Alternate model of deep-learning-based convolutional autoencoder for predicting the output deformations and the coaptation area of the heart valve in the closed state, with the BHV leaflet reference geometry, thickness, and the aortic pressure as input.*

Stats	Euclidean			Hausdorff			Procrustes		
	Max.	Mean	Median	Max.	Mean	Median	Max.	Mean	Median
Training	0.1509	0.0157	0.0115	0.2700	0.0652	0.0574	0.0060	0.0004	0.0003
Validation	0.1346	0.0155	0.0115	0.2568	0.0654	0.0575	0.0056	0.0004	0.0003
Test	0.1477	0.0157	0.0115	0.2689	0.0655	0.0578	0.0059	0.0004	0.0003

Table A3: *Statistics on the metrics describing the prediction of deformations by the modified DLFEA. All units in cm.*

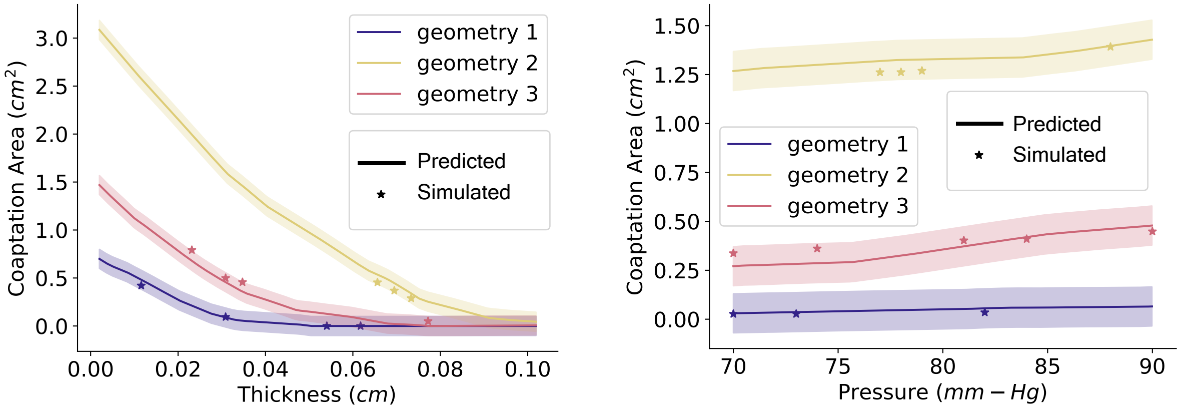

(a) *Thickness* **(b)** *Pressure*

Figure A7: *Figure A7b shows the DLFEA-predicted coaptation area variation with pressure for three specific sets of reference configuration geometries. Figure A7a shows a similar plot with variation in thickness for three specific reference configurations, pressure and other material properties. These plots are generated with 1000 intermediate values of the parameter of interest to get a smooth curve.*

of the training set, with material coefficients fixed at the physiological meaningful values[11]. The corresponding variation of coaptation area with thickness and pressure is shown in Figure A7. Also, we perform similar tests for statistics as the original model and obtain results shown in Figure A8. It can be seen that the trends in the result is similar to the original model with an improvement in correlation for predictions of coaptation area.

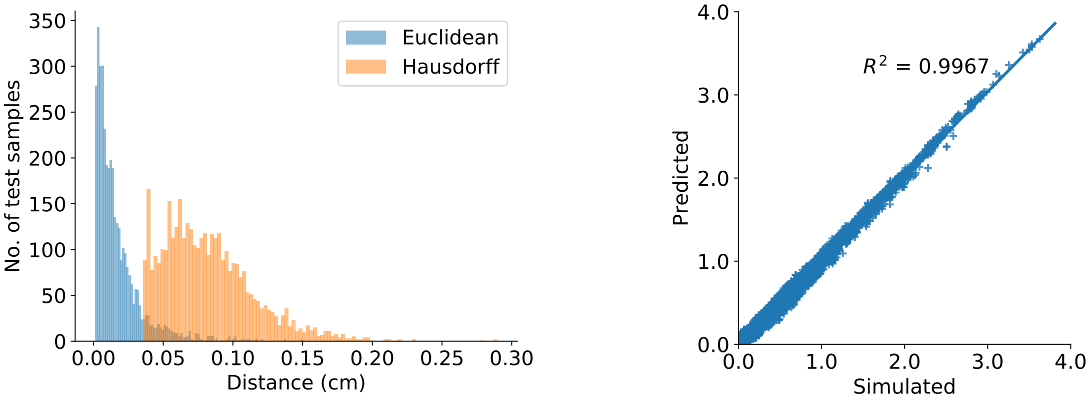

(a) *Distribution of Euclidean and Hausdorff distance*

(b) *Correlation*

Figure A8: *The histograms (Figure A8a) show the Euclidean distance and Hausdorff distance between predicted deformations from DLFEA and the simulated deformations for the test data. Figure A8b shows the DLFEA-predicted coaptation area compared with the coaptation area obtained from simulations. The predicted coaptation area is highly correlated (R = 0.9967) with the simulated values.*

Anecdotal examples

Here, we present another set of anecdotal examples with different configurations (see Figure A9). They represent a more diverse set of examples including large and small deformations. The major deviation between the simulated and the DLFEA predicted deformations occur near the contact region, and particularly, for the cases where the deformations are low (see case 1 and 4 in Figure A9). It can be seen that the DLFEA output is more conservative than the actual simulations.

Extrapolation and interpolation capability of DLFEA

For use in design and diagnostic applications, the DLFEA must be able to accurately predict the deformation for any valve configuration that is obtained by interpolation or extrapolation of the design parameters (up to a reasonable extent). We show some additional results to highlight the extrapolation and interpolation capability of the DLFEA framework in predicting valve deformations. Since, the input geometry to DLFEA is not based on the parametric design variables but the NURBS control points, the DLFEA should be able to predict the deformations for a wide range of valve designs. For this, we perform extropolation experiments by generating samples with different aortic root diameter, which is beyond the sizes of the Aortic root diameter used in training. Also, we change the curvature of the leaflets to the value beyond the range for the training samples. These results correspond to case **a** and **b** shown in Figure A10, where the deviation between the simulated and predicted deformations are less than 10%. In case b, the coaptation area is conservatively predicted to be 0.0 cm^2 by the DLFEA, much lower than the simulated coaptation area value of 0.0281 cm^2. Conservative estimation of the coaptation area can be desirable in valve design applications to cull potential design parameters that might not provide the best performance. In addition to the valve geometry, the thickness and pressure values are also interpolated in case 3, showing that the predicted deformations are still within acceptable error bounds of 10%.

t-SNE analysis

We present some additional t-SNE visualizations that can help us understand how the learnt model can differentiate between the effects of different input parameters on the deformation. In the main text, we presented the t-SNE visualization colored based on the original DLFEA model. Figure A11a shows the data manifold for the modified DLFEA model with particular emphasis on the combination of parameters affecting the data manifold. These inferences are drawn by comparing and analyzing the effect of all variables by labeling each point in the data manifold based on the respective parameter value (see Figure A11b, Figure A11c, Figure A11d, Figure A11e, Figure A11f). The figure shows that certain clusters in the manifold have similar parameter value, while some clusters have a gradual variation of that parameter value. In addition, there are also interaction between the different parameters. For example, the valve thickness

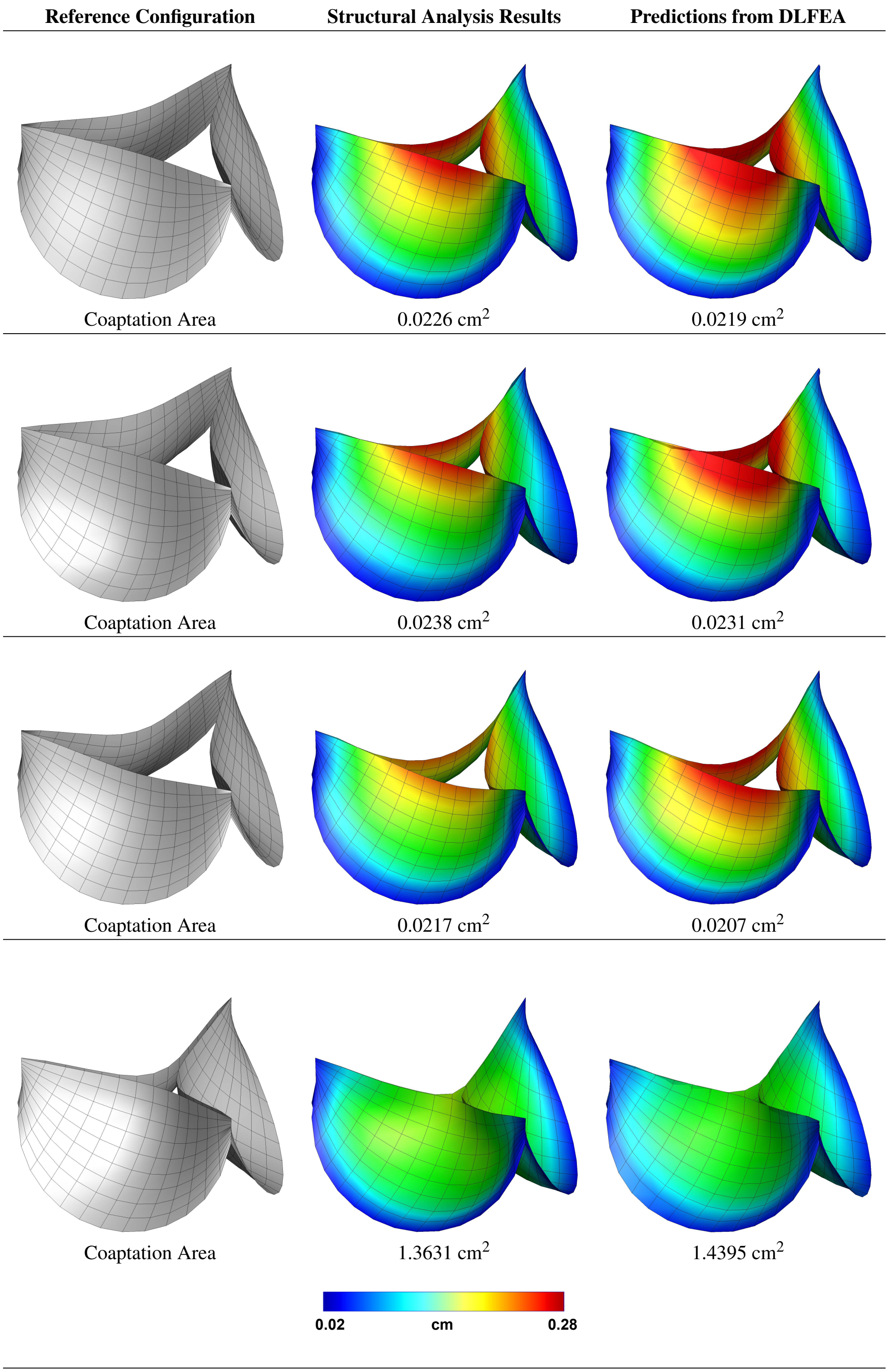

Figure A9: *Illustrative examples of the valve deformations obtained from isogeometric analysis and predicted by the DLFEA framework. The simulated and predicted coaptation area is also shown below the deformations. The color in the image depicts the absolute value of the displacement in the deformed configuration of the bioprosthetic heart valve.*

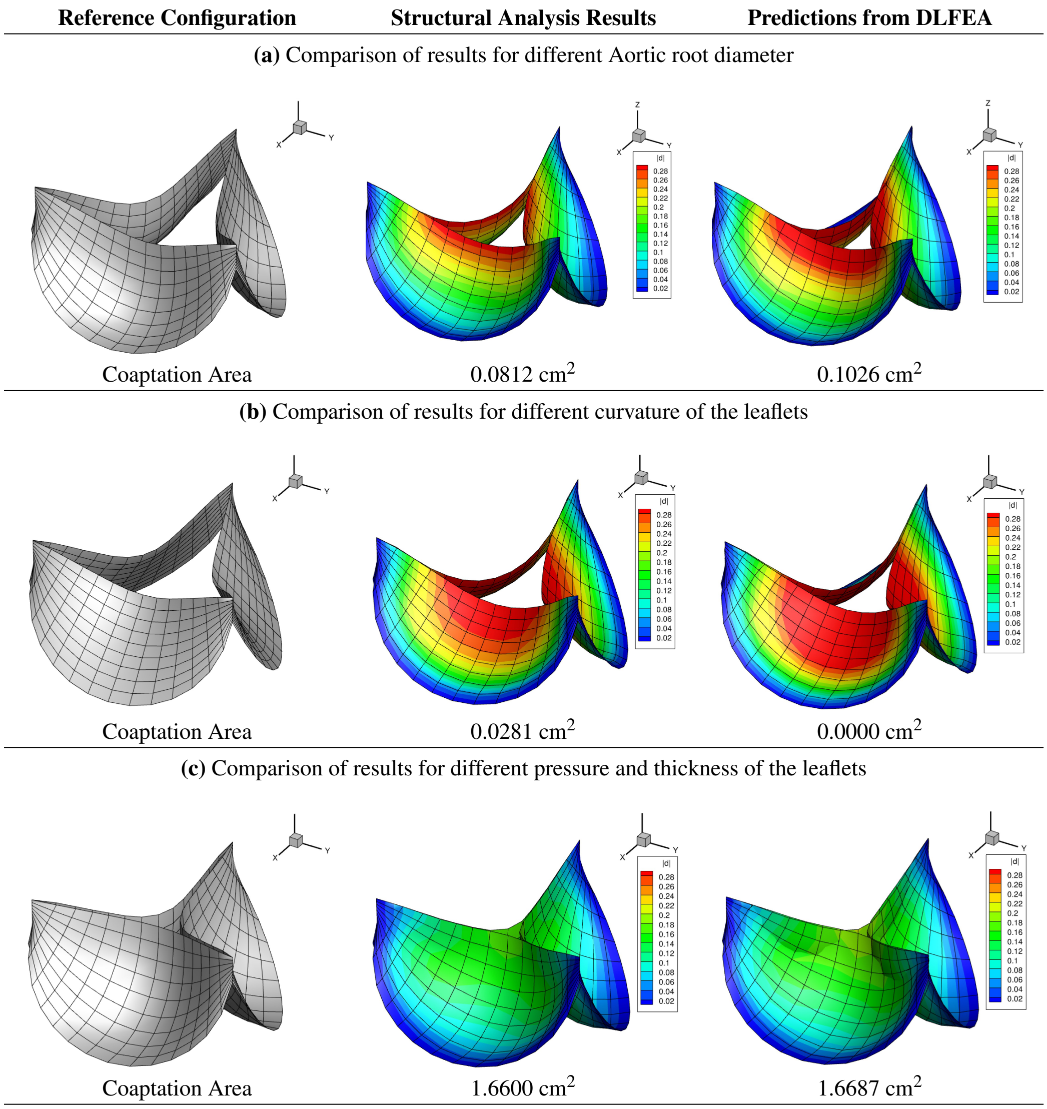

Figure A10: *Generalization capability of DLFEA to predict deformations with different input parameters. The color in the image depicts the absolute value of the displacement in the deformed configuration of the bioprosthetic heart valve.*

and free-edge curvature values interact; high free-edge curvature and high thickness values correlate and form a single cluster while this interaction is not significant when the free-edge curvature value is very low. Similarly, the Aortic pressure also interacts with other parameter values with low aortic pressure, low valve thickness, and high free-edge height forming a cluster. On further examination, we observe that this cluster belongs to very low or zero coaptation area. These correlation between the variables and the formation of clusters in t-SNE visualizations show that DLFEA captures the effect of each parameter on the deformations and coaptation area.

Results video

We have attached a video demonstrating the key aspects of this work. Specifically, it contains a demo of parametric design of bioprosthetic heart valves, which is discussed in detail in the first section of the Supplement. Further, we show a demonstration of a valve deformation simulation using traditional IGA and using our DLFEA framework. Finally, we visually compare the results obtained from both IGA and DLFEA.

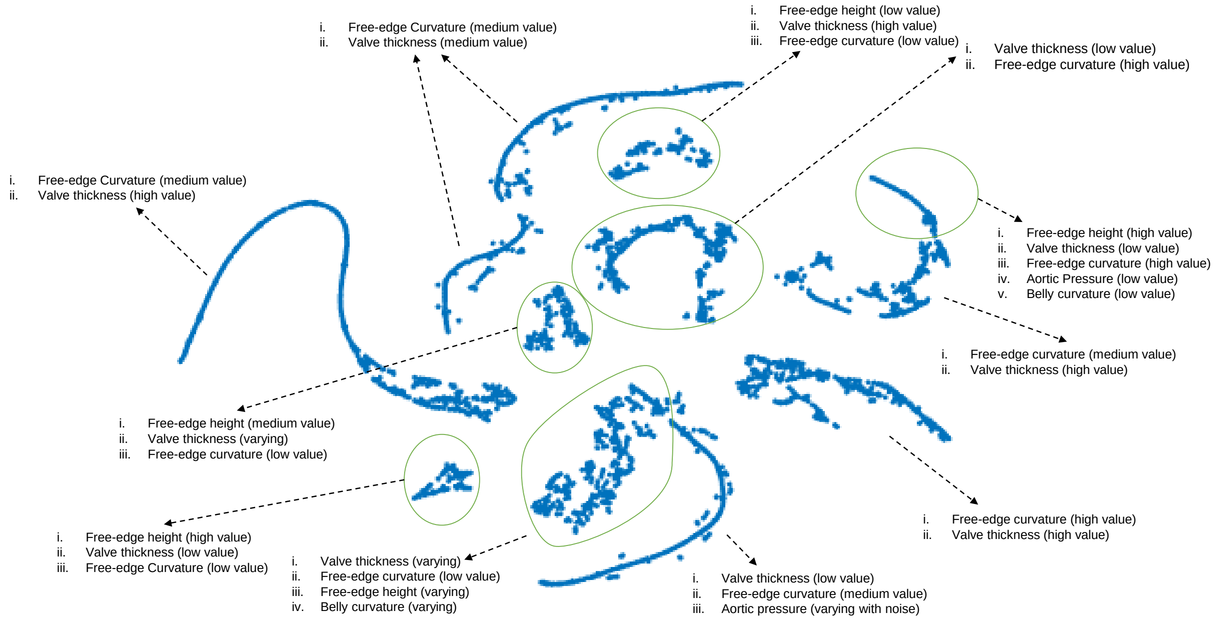

(a) *t-SNE*

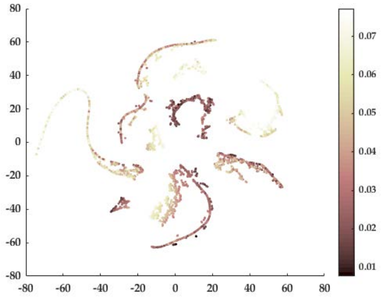

(b) *Valve thickness*

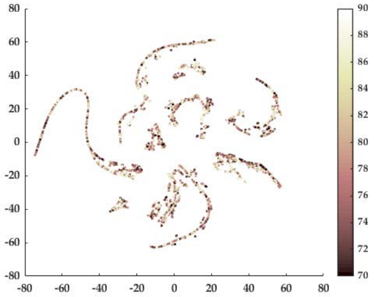

(c) *Aortic pressure*

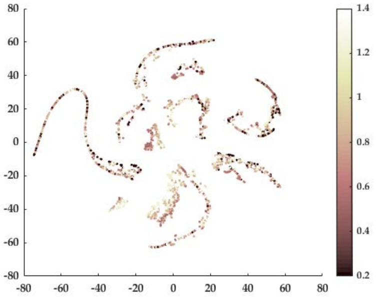

(d) *Belly curve parameter*

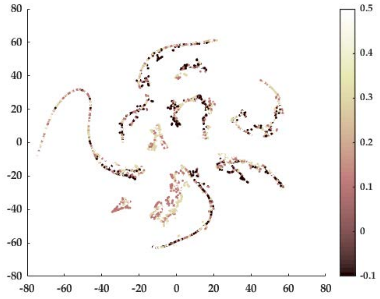

(e) *Free-edge height*

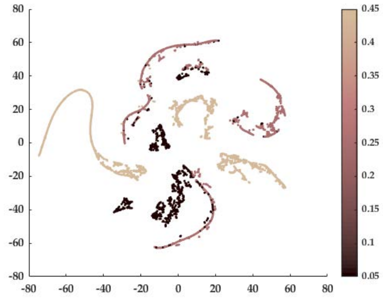

(f) *Free-edge curve parameter*

Figure A11: *t-distributed stochastic neighborhood embedding (t-SNE) of the higher dimensional manifold colored by the different parameters learnt by DLFEA. t-SNE generates a lower dimensional embedding of the data using the learnt model, which can provide insights into the distribution of the data.*

References

[1] Fred L Bookstein. *Morphometric tools for landmark data: Geometry and Biology*. Cambridge University Press, 1997.

[2] Andrés Caballero, Fatiesa Sulejmani, Caitlin Martin, Thuy Pham, and Wei Sun. Evaluation of transcatheter heart valve biomaterials: biomechanical characterization of bovine and porcine pericardium. *Journal of the mechanical behavior of biomedical materials*, 75:486–494, 2017.

[3] Sambit Ghadai, Aditya Balu, Soumik Sarkar, and Adarsh Krishnamurthy. Learning localized features in 3D CAD models for manufacturability analysis of drilled holes. *Computer Aided Geometric Design*, 62:263–275, 2018.

[4] T. J. R. Hughes, John A Cottrell, and Yuri Bazilevs. Isogeometric analysis: CAD, finite elements, NURBS, exact geometry and mesh refinement. *Computer Methods in Applied Mechanics and Engineering*, 194(39-41):4135–4195, 2005.

[5] Josef Kiendl, Ming-Chen Hsu, Michael CH Wu, and Alessandro Reali. Isogeometric kirchhoff–love shell formulations for general hyperelastic materials. *Computer Methods in Applied Mechanics and Engineering*, 291:280–303, 2015.

[6] Alex Krizhevsky, Ilya Sutskever, and Geoffrey E Hinton. Imagenet classification with deep convolutional neural networks. In F. Pereira, C. J. C. Burges, L. Bottou, and K. Q. Weinberger, editors, *Advances in Neural Information Processing Systems 25*, pages 1097–1105. Curran Associates, Inc., 2012.

[7] Yann LeCun, Léon Bottou, Yoshua Bengio, and Patrick Haffner. Gradient-based learning applied to document recognition. *Proceedings of the IEEE*, 86(11):2278–2324, 1998.

[8] S Morganti, F Auricchio, DJ Benson, FI Gambarin, S Hartmann, T. J. R. Hughes, and A Reali. Patient-specific isogeometric structural analysis of aortic valve closure. *Computer Methods in Applied Mechanics and Engineering*, 284:508–520, 2015.

[9] LA Piegl and Wayne Tiller. The NURBS book, 1997.

[10] S. C. Wong, A. Gatt, V. Stamatescu, and M. D. McDonnell. Understanding data augmentation for classification: When to warp? In *2016 International Conference on Digital Image Computing: Techniques and Applications (DICTA)*, pages 1–6, Nov 2016.

[11] Michael CH Wu, Rana Zakerzadeh, David Kamensky, Josef Kiendl, Michael S Sacks, and Ming-Chen Hsu. An anisotropic constitutive model for immersogeometric fluid–structure interaction analysis of bioprosthetic heart valves. *Journal of biomechanics*, 74:23–31, 2018.

[12] Fei Xu, Simone Morganti, Rana Zakerzadeh, David Kamensky, Ferdinando Auricchio, Alessandro Reali, T. J. R. Hughes, Michael S Sacks, and Ming-Chen Hsu. A framework for designing patient-specific bioprosthetic heart valves using immersogeometric fluid–structure interaction analysis. *International Journal for Numerical Methods in Biomedical Engineering*, 34(4):e2938, 2018.
